# Human α‐synuclein overexpression upregulates SKOR1 in a rat model of simulated nigrostriatal ageing

**DOI:** 10.1111/acel.14155

**Published:** 2024-03-26

**Authors:** Noelia Morales‐Prieto, Rebekah Bevans, Adam O'Mahony, Aaron Barron, Conor Giles Doran, Erin McCarthy, Ruth M. Concannon, Susan R. Goulding, Cathal M. McCarthy, Louise M. Collins, Aideen M. Sullivan, Gerard W. O'Keeffe

**Affiliations:** ^1^ Department of Anatomy and Neuroscience, School of Medicine University College Cork Ireland; ^2^ Department of Pharmacology and Therapeutics, School of Medicine University College Cork Cork Ireland; ^3^ Department of Physiology, School of Medicine University College Cork Cork Ireland; ^4^ Parkinson's Disease Research Cluster (PDRC) University College Cork Cork Ireland; ^5^ APC Microbiome Ireland University College Cork Cork Ireland

**Keywords:** ageing, microarray, mitochondria, nigrostriatal, SKOR1, α‐Synuclein

## Abstract

Parkinson's disease (PD) is characterised by progressive loss of dopaminergic (DA) neurons from the *substantia nigra* (SN) and α‐synuclein (αSyn) accumulation. Age is the biggest risk factor for PD and may create a vulnerable pre‐parkinsonian state, but the drivers of this association are unclear. It is known that ageing increases αSyn expression in DA neurons and that this may alter molecular processes that are central to maintaining nigrostriatal integrity. To model this, adult female Sprague–Dawley rats received a unilateral intranigral injection of adeno‐associated viral (AAV) vector carrying wild‐type human αSyn (AAV‐αSyn) or control vector (AAV‐Null). AAV‐αSyn induced no detrimental effects on motor behaviour, but there was expression of human wild‐type αSyn throughout the midbrain and ipsilateral striatum at 20 weeks post‐surgery. Microarray analysis revealed that the gene most‐upregulated in the ipsilateral SN of the AAV‐αSyn group was the SKI Family Transcriptional Corepressor 1 (SKOR1). Bioenergetic state analysis of mitochondrial function found that SKOR1 overexpression reduced the maximum rate of cellular respiration in SH‐SY5Y cells. Furthermore, experiments in SH‐SY5Y cells revealed that SKOR1 overexpression impaired neurite growth to the same extent as αSyn, and inhibited BMP‐SMAD‐dependent transcription, a pathway that promotes DA neuronal survival and growth. Given the normal influence of ageing on DA neuron loss in human SN, the extent of αSyn‐induced SKOR1 expression may influence whether an individual undergoes normal nigrostriatal ageing or reaches a threshold for prodromal PD. This provides new insight into mechanisms through which ageing‐related increases in αSyn may influence molecular mechanisms important for the maintenance of neuronal integrity.

## Graphical abstract

Ageing increases α‐synuclein (αSyn) in midbrain dopaminergic neurons, which may alter molecular processes central to maintaining nigrostriatal integrity. Here we show that intranigral overexpression of αSyn increased Skor1 expression which reduced the maximum rate of cellular respiration and neurite growth in vitro. This provides new insight into mechanisms through which ageing‐related increases in αSyn may influence molecular mechanisms important for the maintenance of neuronal integrity.

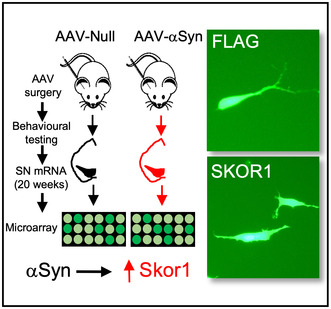

**Abbreviations**
AAV: adeno‐associated viral vector
BMP: bone morphogenetic protein
DA: dopaminergic
DAT: dopamine transporter
PD: Parkinson's disease
RLS: restless leg syndrome
SKOR1: SKI Family Transcriptional Corepressor 1
SN: *Substantia nigra*
TH: tyrosine hydroxylase
αSyn: α‐synuclein

## 1 INTRODUCTION, RESULTS AND DISCUSSION

Parkinson's disease (PD) is the fastest‐growing neurological disorder (Dorsey et al., 2018; Group, 2017). It is a neurodegenerative disease characterised by the progressive degeneration of midbrain dopaminergic (DA) neurons in the *substantia nigra* (SN), which form the nigrostriatal tract (Bloem et al., 2021; Kordower et al., 2013). Ageing is the biggest risk factor for PD, which affects 1% of people over the age of 60, rising to 5% of those over the age of 85. Despite the wealth of epidemiological evidence for ageing as the primary risk factor for PD, the biological drivers of this association are largely unknown. However, it is thought that SN neuronal loss occurs in humans as a result of ageing, at a rate of approximately 7% per decade (Stark & Pakkenberg, 2004). This is supported by imaging studies in humans showing that ageing of the nigrostriatal tract begins in the third decade and progresses throughout life until old age, when it exhibits significant degeneration (Seo & Koo, 2021). In agreement, a study in non‐human primates has shown that ageing‐related changes in the dopamine system actively create a vulnerable pre‐parkinsonian state (Collier et al., 2017). The drivers of these changes are unknown, but a leading candidate is ageing‐related change in α‐synuclein (αSyn) expression.

Misfolded αSyn is a major component of Lewy bodies, the intracellular proteinaceous inclusions that characterise PD pathology (Braak et al., 2003, 1999; Spillantini et al., 1998, 1997). Moreover, mutations (Kruger et al., 1998; Polymeropoulos et al., 1997), or duplications and triplications (Nishioka et al., 2006; Singleton et al., 2003), in the αSyn gene, *SNCA*, cause autosomal‐dominant PD. αSyn is a normal synaptic protein that displays a change in intraneuronal localisation as a result of ageing (Chu & Kordower, 2007; Collier et al., 2017). Ageing leads to a prominent increase in αSyn staining within the soma of SN neurons, which is correlated with reduced expression of phenotypic markers of DA neurons (Chu & Kordower, 2007; Collier et al., 2017). This suggests that age‐related increases in intraneuronal αSyn expression may alter gene expression in the SN, thus affecting molecular processes that are central to maintaining nigrostriatal integrity.

**FIGURE 1:**
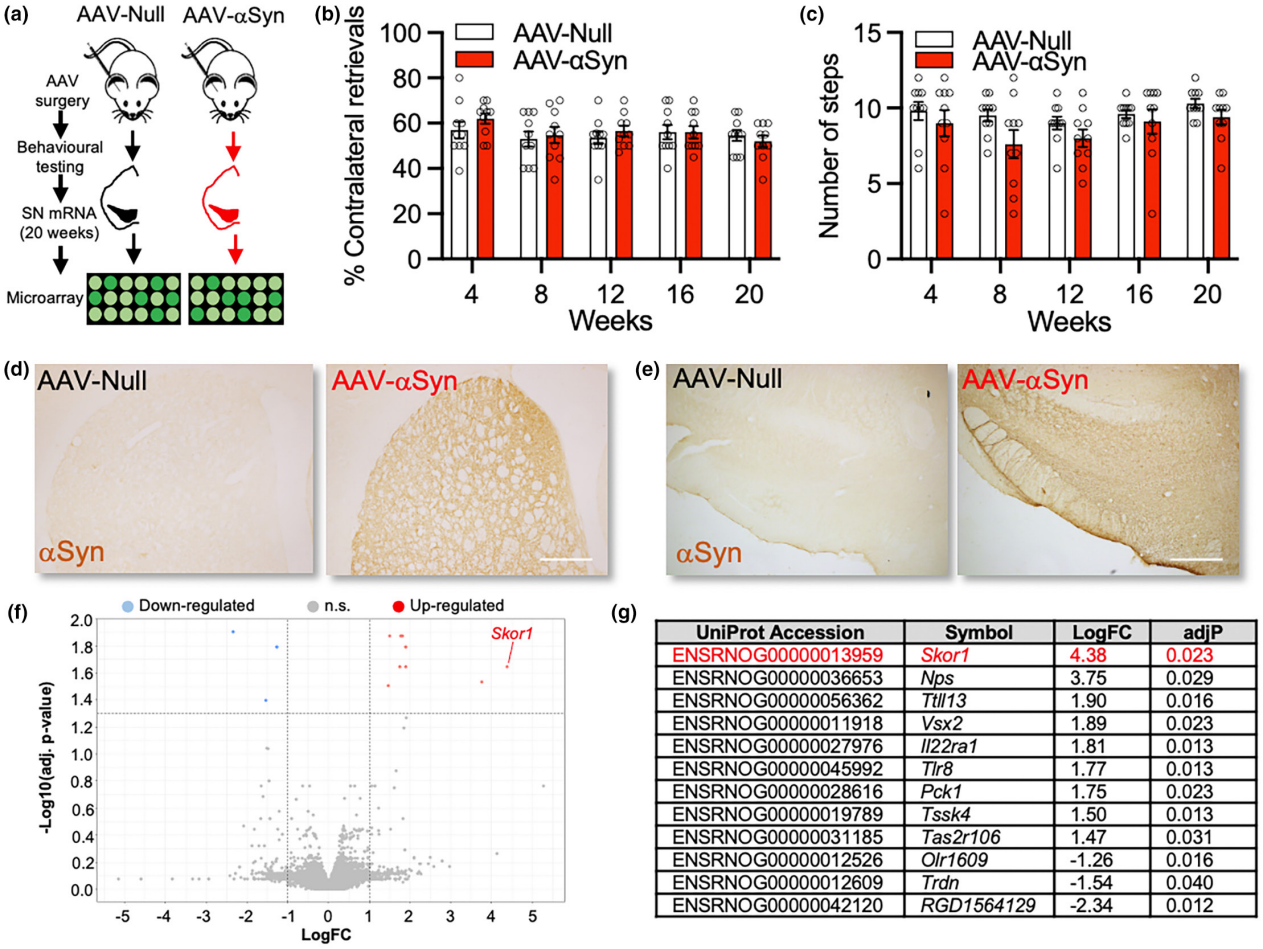
Overexpression of wild‐type human α‐synuclein in the rat *substantia nigra* upregulates *Skor1*. (a) Schema of the experimental design. (b, c) Graphs showing the results of (b) the corridor test and (c) the stepping test at 4–20 weeks post‐surgery. (d, e) Representative images showing wild‐type human α‐synuclein (αSyn) immunostaining in (d) striatum and (e) SN at 20 weeks after unilateral stereotaxic injection of either an AAV‐Null or AAV‐αSyn vector into the SN. Scale bars = 40 μm. (f) Volcano plot (adjusted *p*‐value vs. log fold‐change) and (g) list of the genes overexpressed and under‐expressed (following a Benjamini–Hochberg multiple‐testing correction) in the SN, in the AAV‐αSyn compared to the AAV‐Null group at 20 weeks after unilateral stereotaxic injection of either an AAV‐Null or AAV‐αSyn vector into the SN. Microarray analysis was performed on *n* = 3 per group. *Skor1* was the top‐ranked overexpressed gene in the AAV‐αSyn group (logFC = 4.38, Benjamini–Hochberg adjusted *p* = 0.023). n.s, not significant.

To determine the effects of overexpression of wild‐type human αSyn on the transcriptome of a rat model of nigrostriatal ageing, adult female Sprague–Dawley rats received a unilateral intranigral injection of an adeno‐associated viral (AAV) vector carrying the wild‐type human αSyn transgene (AAV‐αSyn), or a null control vector (AAV‐Null) (Figure 1a). This has been shown to lead to 36% reduction in TH‐immunoreactive, and ∼30% reduction in DAT‐immunoreactive, neurons at 20 weeks post‐surgery (Goulding et al., 2021). Although estimates vary, it is thought that the motor symptoms of PD do not manifest until ∼60% of SN DA neurons have been lost (Cheng et al., 2010). In agreement, we found no significant effect of αSyn in the corridor (F_(1,9)_ = 0.5606, *p* = 0.473) (Figure 1b) or stepping (F_(1,9)_ = 3.782, *p* = 0.084) (Figure 1c) tests of sensorimotor function at 4, 8, 12, 16 and 20 weeks post‐surgery. Immunohistochemistry for human wild‐type αSyn on sections of the midbrain and striatum showed that αSyn was expressed throughout the ipsilateral striatum (Figure 1d) and SN (Figure 1e). To confirm if the increase in αSyn resulted in reduced expression of phenotypic markers of DA neurons (Chu & Kordower, 2007; Collier et al., 2017), we examined the expression of 84 PD‐relevant genes in the ipsilateral SN, using the PD RT^2^ Profiler PCR Array. The AAV‐αSyn group had significant reductions in the expression of transcripts for genes involved in the dopamine biosynthesis pathway, including *Th*, *Slc6a3* (*Dat1*) and *Ddc* (*dopa decarboxylase*), as well as reductions in *Park7* (Figure S1). This model recapitulates features of an aged nigrostriatal system in that there is increased expression of αSyn and reduced expressed of phenotypic markers of DA neurons, but not to such a degree that leads to motor impairments.

We next investigated global transcriptome changes in the ipsilateral SN. cRNA was prepared from the mRNA extracted from the ipsilateral SN and hybridised to oligonucleotide microarrays. Of the 39,956 sequences represented on the array, 2608 of these were found to be differentially expressed between the AAV‐Null and the AAV‐αSyn group (*p* < 0.05), before a multiple testing correction was applied (data not shown). This included genes found in the PCR array, including *Th* (*p* = 0.0043), *Slc6a3* (*p* = 0.0122) and *Ddc* (*p* = 0.0198). Of the *n* = 2608 sequences, *n* = 21 remained statistically significant after a multiple testing correcting using the Benjamini–Hochberg method (adj*p* < 0.05) (Benjamini & Hochberg, 1995). These 21 sequences mapped to 12 unique gene identifiers. The gene most upregulated in the αSyn group was the SKI Family Transcriptional Corepressor 1 (Skor1) (Log FC = 4.3821; adj*p* = 0.0225) (Figure 1f,g). SKOR1 is highly expressed in the CNS of humans and mice. It plays a regulatory role in the transcription of genes that are involved in pathways related to restless legs syndrome (RLS) (Sarayloo et al., 2020), and variants in SKOR1 have been associated with RLS (Jimenez‐Jimenez et al., 2018).

**FIGURE 2:**
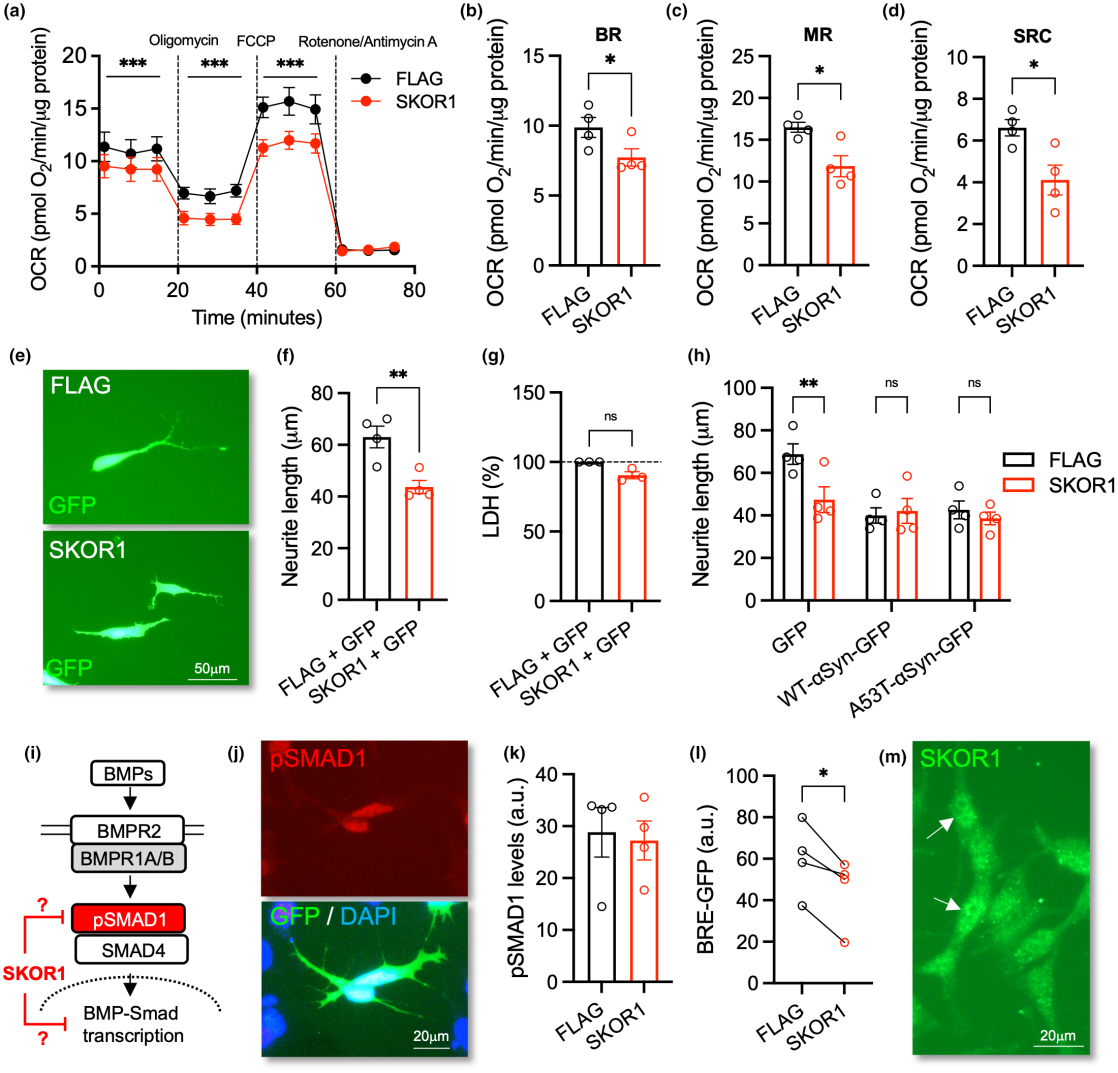
Overexpression of SKOR1 impairs neurite growth, mitochondrial function and BMP‐SMAD signalling in SH‐SY5Y cells. (a) Oxygen consumption rate (OCR) on the Seahorse XF Mito Stress Test in SH‐SY5Y cells at 72 h post‐transfection with 500 ng of a plasmid expressing either FLAG or FLAG‐tagged SKOR1. (b–d) The graphs of individual parameters of respiration showing (b) basal respiration (BR), (c) maximal respiration (MR) and (d) spare respiratory capacity (SRC). Data are mean ± SEM of OCR values normalised to protein content per well from *n* = 4 independent experiments. Mixed effects model with either (a) post‐hoc Bonferroni test (***p < 0.001), or (b–d) Student's *t*‐test (*p < 0.05, ***p < 0.001). (e) Representative photomicrographs and (f) the graphs of neurite length and (g) LDH assay of SH‐SY5Y cells at 72 h post transfection with 500 ng of a plasmid expressing either FLAG or FLAG‐tagged SKOR1 plus GFP to visualise transfected cells. (h) The graph of neurite length of SH‐SY5Y cells at 72 h post transfection with 500 ng of a plasmid expressing either FLAG or FLAG‐tagged SKOR1 with either GFP or GFP‐tagged wild‐type α‐synuclein (WT‐αSyn) or GFP‐tagged A53T‐αSyn. (e–h) Data are mean+/‐SEM from *n* = 4 independent experiments. Student's *t*‐test. (**p < 0.01). (i) Schema of the BMP‐Smad signalling pathway. (j) Representative photomicrographs showing pSMAD1 staining, and graphs showing (k) pSMAD1 levels and (l) GFP fluorescence in SH‐SY5Y cells at 72 h post‐transfection with 500 ng of a plasmid expressing either FLAG or FLAG‐tagged SKOR1 plus (k) GFP plasmid or (l) with a reporter plasmid expressing GFP under the control BMP‐responsive element (BRE). (m) Immunocytochemistry showing the cellular localisation of SKOR1 (green) in SH‐SY5Y cells. Scale bar = 20 μm. Data are mean+/‐SEM from *n* = 4 independent experiments. (k, l) Paired *t*‐test (*p < 0.05).

We next hypothesised that if SKOR1 is involved in αSyn‐mediated cellular changes, then SKOR1 overexpression should mimic the effects of αSyn on cellular function. To test this hypothesis, we used two functional readouts that are known to be negatively affected by αSyn: mitochondrial function (Di Maio et al., 2016; McCarthy et al., 2022) and neurite growth (Goulding et al., 2019; Mazzocchi et al., 2020). To investigate mitochondrial function in SH‐SY5Y cells expressing FLAG or SKOR1, we analysed cellular bioenergetic state using the Agilent Seahorse XF platform. Like that seen in cells overexpressing αSyn (Di Maio et al., 2016; McCarthy et al., 2022), a consistent reduction in OCR was observed in SKOR1‐expressing cells, relative to the FLAG controls (Figure 2a). We also examined individual parameters of respiration and found significant reductions in several parameters in the SKOR1 group, including maximal respiration (Figure 2b–d). Maximal respiration induced by the addition of FCCP mimics a physiological energy demand by stimulating mitochondria to operate at maximum capacity (Brand & Nicholls, 2011). These data show that SKOR1 overexpression reduces the maximum rate of respiration that a cell can achieve.

We next used neurite outgrowth as a second functional readout to determine if SKOR overexpression mimics the inhibitory effect of αSyn on neurite growth, which has previously been shown in SH‐SY5Y cells (Goulding et al., 2019; Mazzocchi et al., 2020), rodent neurons overexpressing αSyn (Koch et al., 2015), and human iPSC‐derived DA neurons carrying *SNCA* mutations, duplications or triplications (Kouroupi et al., 2017; Oliveira et al., 2015). We found that SKOR1 overexpression impaired neurite growth in SH‐SY5Y cells expressing FLAG or SKOR1 (Figure 2e,f), but did not adversely affect cell viability (Figure 2g). We repeated this experiment with or without co‐transfection with plasmids expressing GFP‐tagged WT or A53T‐αSyn. SKOR1 had the same inhibitory effect on neurite growth in cells co‐transfected with WT‐αSyn or A53T‐αSyn, and did not exacerbate reductions in neurite growth induced by either (Figure 2h). This is consistent with SKOR1 being a downstream mediator of the detrimental effects of αSyn on neurite growth.

We next investigated the cellular mechanisms underpinning the effects of SKOR1 overexpression on neurite growth. Interestingly, SKOR1 has been reported to be a negative regulator of the BMP‐Smad pathway in HEK‐293 T cells (Fischer et al., 2012) (Figure 2i). Two members of the bone morphogenetic protein family, growth differentiation factor 5 (GDF5; also known as BMP14) (Goulding et al., 2021) and BMP5/7 (Vitic et al., 2021), have been shown to prevent αSyn‐induced DA degeneration in vivo. Moreover, activation of this pathway promotes neurite growth in SH‐SY5Y cells (Hegarty et al., 2013) and rodent DA neurons (Hegarty et al., 2014). To investigate this further, we examined cellular levels of phospho‐SMAD1 and found that, while SKOR1 overexpression did not affect these (Figure 2i,j), it significantly inhibited BMP‐dependent transcription in SH‐SY5Y cells transfected with a BMP‐responsive element (BRE)‐GFP reporter construct. The effect of SKOR1 on BMP‐SMAD‐dependent transcription is consistent with the predominantly‐nuclear localisation of SKOR1 in SH‐SY5Y cells (Figure 2m). Since BMP‐Smad signalling has also been shown to enhance mitochondrial activity (Li et al., 2022), it is possible that SKOR1‐induced impairments in the BMP‐SMAD pathway (Figure 2m) may contribute to SKOR1‐induced alterations in both mitochondrial function and neurite growth.

It is interesting to note in a wider context that SMAD1 was one of three transcription factors found be downregulated in blood samples from PD patients (Tan et al., 2018), while there was increased expression of the BMP inhibitory SMAD6 in the PD SN (Vitic et al., 2021). Furthermore, a gene ontology analysis of transcriptome from human SN reported alterations in the BMP‐SMAD pathway in Braak stage 1–2 PD subjects in comparison to controls (Dijkstra et al., 2015). It is worth noting that, while known genetic variants in SKOR1 are associated with RLS (Jimenez‐Jimenez et al., 2018), these variants are not linked to PD risk, although one marker, rs12593813, in SKOR1 has been associated with a high frequency of tremor in PD patients (Gan‐Or et al., 2015). This suggests a possible effect of SKOR1 on the motor system, but this requires further investigation.

In future work, it will be important to confirm these findings in males, as only female rats were used in the current study for logistic reasons. It will also be crucial to validate our findings at the protein level, and to determine the precise role, if any, of SKOR1 in DA degeneration in vivo. Additionally it is difficult to extrapolate the short‐term overexpression of SKOR1 in vitro to the in vivo scenario; however, these data suggest a model in which age‐related increases in αSyn expression in DA neurons in the SN (Chu & Kordower, 2007; Collier et al., 2017) lead to increased cellular levels of SKOR1, which inhibit the BMP‐SMAD signalling pathway during normal ageing. Given the natural variation in DA neuron number in healthy humans (von Linstow et al., 2020), and the age‐related 7% per decade loss of these cells in the human SN (Stark & Pakkenberg, 2004), it is possible that the extent to which αSyn upregulates SKOR1 expression may influence the process of normal nigrostriatal ageing, perhaps through the modulation of mitochondrial function and axonal integrity, thereby exacerbating an individual's risk of developing PD due to age.

## AUTHOR CONTRIBUTIONS

NMP and CGD generated, analysed and curated the transcriptome data. RB, AOM performed the in vitro experiments. AB analysed mitochondrial function. NMP and EM performed the PCR array. RMC and SRG performed the surgery and behavioural testing. CMM, LMC, AMS and GOK supervised the work. NMP, AMS and GOK wrote the manuscript. All authors contributed to reviewing and editing of the final manuscript.

## FUNDING INFORMATION

Work in the authors laboratories is supported by Science Foundation Ireland (Grant 19/FFP/6666), Cure Parkinson's (Grant CP:GO01) and the European Union's Horizon 2020 Research and Innovation programme under the Marie Skłodowska‐Curie grant agreement No 890290.

## CONFLICT OF INTEREST STATEMENT

All authors declare no conflicts of interest.

## Supporting information


Figure S1.

Data S1.

Data S2.

## Data Availability

All data are either publicly available (GSE252918) or are available from the corresponding authors upon reasonable request.
