# Supplementary figures and images for: Human α‐synuclein overexpression upregulates SKOR1 in a rat model of simulated nigrostriatal ageing
Source: Aging Cell. 2024 Mar 26;23(6):e14155. doi: 10.1111/acel.14155 (PMC11296121; doi:10.1111/acel.14155)

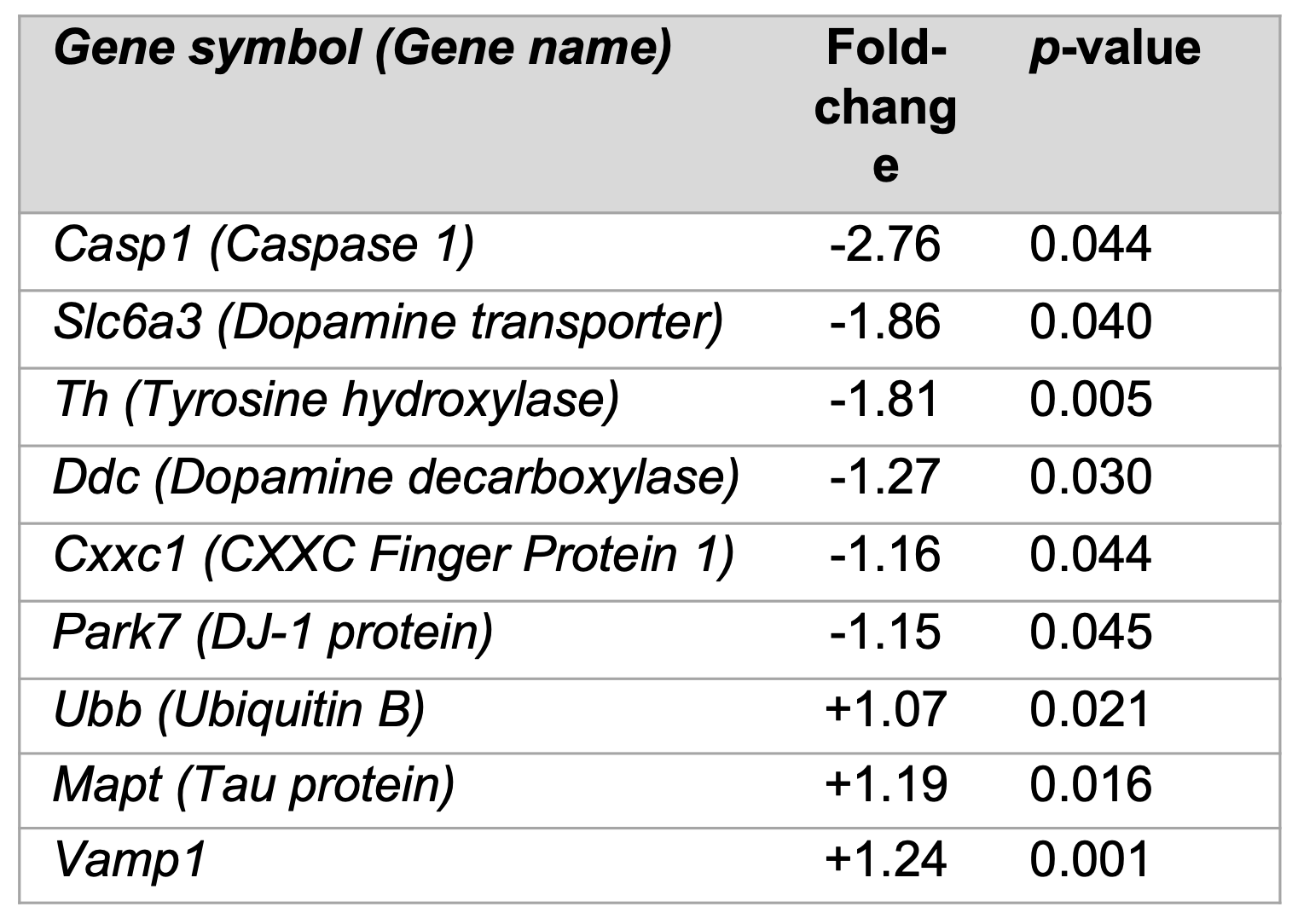

Supplement: Supplementary file 1 — Figure S1. [file ACEL-23-e14155-s002.tif]
