# Supplementary material for: Human α‐synuclein overexpression upregulates SKOR1 in a rat model of simulated nigrostriatal ageing
Source: Aging Cell. 2024 Mar 26;23(6):e14155. doi: 10.1111/acel.14155 (PMC11296121; doi:10.1111/acel.14155)
Supplement: Supplementary file 2 — Data S1. [file ACEL-23-e14155-s001.docx]

**Materials and Methods**

**Animal husbandry and study design**

Three-month-old adult female Sprague‐Dawley rats (Envigo, UK) were housed on a 12h:12h light:dark cycle with *ad libitum* access to food and water. Animals were assigned to either an AAV-Null (*n*=10) or AAV-αSyn (n=10) group and received unilateral stereotaxic injection of either AAV6-Null (5.3×10^9^ vg/µL) or AAV6-αSyn (5.2×10^10^ vg/µL) (Vector Biosystems Inc) into the SN at coordinates: AP ‐5.3, ML ± 2.0, DV ‐7.2 relative to bregma. Post-surgery, animals received the analgesic Carprofen (5 mg/kg, s.c.) and 5% glucose solution (i.p.) and were allowed to fully recover on a heating mat. Rats were housed in groups of four in standard home cages with environmental enrichment. The Corridor test, a drug-free behavioural test that assesses lateralized sensorimotor neglect (Dowd et al., 2005), and the Stepping test, which measures forelimb akinesia (Olsson et al., 1995), were performed at 4, 8, 12, 16 and 20 weeks post-surgery. These tests have been shown to detect αSyn-induced impairments in motor function (Naughton et al., 2017; Negrini et al., 2022). For behavioural analysis, ten animals from each experimental group were used. All experiments were in accordance with the European Directive 2010/63/EU and under an authorisation granted by the Health Products Regulatory Authority Ireland (AE19130/P057).

**Tissue processing and immunohistochemistry**

At 20 weeks post-surgery, animals were euthanised by decapitation under isoflurane anaesthesia (5% in O_2_) for qRT-PCR and microarray analyses, or by transcardial perfusion with 4% paraformaldehyde under terminal pentobarbital anaesthesia (50 mg/kg) for immunohistochemistry. For qRT-PCR and microarray analysis, the SN was microdissected from each brain and stored at -80 ºC until use. For immunohistochemistry, brains were post-fixed in 4% paraformaldehyde for 24 h and cryoprotected in 30% sucrose solution with 0.1% sodium azide. Thirty-micrometre (30 µm) sections were cut on a freezing-stage sledge microtome and immunohistochemistry was carried out as previously described (Goulding et al. 2021) using a primary antibody to α-Syn (1:1000, Millipore). Images of the sections were taken using the Olympus BX53 Upright Microscope.

**Microarray analysis**

For transcriptome analysis, three biological replicates of each experimental group were used. Total RNA was isolated using the RNeasy Plus Universal Tissue Kit (QIAGEN) following the manufacturer's recommendations, and quantified using a NanoDrop™ spectrophotometer. Three micrograms of RNA from each individual sample were sent to Centro de Investigación Príncipe Felipe (Valencia, Spain) and its integrity was assessed using a 4200 TapeStation (Agilent Technologies, Inc.). Gene expression microarray analyses were performed according to the Agilent protocol for One-Color Microarray-Based Gene Expression Analysis. Cyanine-3 (Cy3) labelled cRNA was prepared from 2 µg RNA using the Agilent Low-Input QuickAmp Labelling Kit (Agilent) according to manufacturer’s instructions, followed by RNeasy column purification (QIAGEN^®^). Dye incorporation and cRNA yield was then checked with a NanoDrop™ spectrophotometer. 600 µg of Cy3-labelled cRNA was then fragmented and hibridized to the SurePrint G3 Rat Gene Expression Microarray v2 G4858A-074036 8x60K at 65 °C for 17 h in a rotating Agilent G2545A Hybridization Oven, using the Agilent Gene Expression Hybridization Kit. After washing and drying in an ozone-free atmosphere, the slides were scanned with an Agilent Microarray Scanner (G2565C) using one colour scan setting for 8x60k array slides. Agilent Feature Extraction Software 11.5.1.1 was used for image analysis and quantification of fluorescence data. Microarray data was deposited in the Gene Expression Omnibus (GEO) platform (<https://www.ncbi.nlm.nih.gov/geo/>) with accession number GSE252918. Data analyses was performed with the *limma* package 3.30.7 from the Bioconductor Project for differential expression analysis. After removal of experimental artifacts, Dark and GE_Bright corners and under-threshold intensity spots, the data were normalized by quantile normalization. Finally, differential expression analysis was carried out with the *decideTests* function of *limma.*

**PCR-array**

Total RNA from each ipsilateral SN region was isolated with the RNeasy Plus Universal Mini Kit (QIAGEN®) following the manufacturer’s indications. RNA integrity was checked by agarose-bleach gel (Aranda, LaJoie and Jorcyk, 2012). RNA concentration was determined by measuring absorbance at 260/280 nm in a NanoDrop™ spectrophotometer. cDNA was synthetized with the RT^2^ First Strand Kit (QIAGEN®). Samples were analysed using the Rat Parkinson's Disease RT² Profiler PCR Array (GeneGlobe ID - PARN-124Z, QIAGEN®). Amplification of cDNA was carried out according to manufacturer’s indications using the RT^2^ SYBR® Green qPCR Mastermix (QIAGEN®) in a LightCycler® 96 System (Roche Molecular Systems, Inc.). After a 10-min denaturalization step at 95 °C, 40 cycles of amplification were carried out (15s 95 °C, 60s 60 °C). Cq values were analysed at QIAGEN’S GeneGlobe Data Analysis Center.

**Seahorse assay to assess mitochondrial function**

Assessment of mitochondrial function was carried out as previously described (McCarthy et al., 2022). Briefly, SH-SY5Y cells were plated at a density of 4 x 10^5^ cells/well in a XF96 culture plate and were transfected after 24h with 500 ng of plasmid expressing either FLAG or FLAG-tagged SKOR1. Mitochondrial function was assessed at 72h after transfection. At 1h before the assay, the media was changed to Seahorse XF DMEM media, supplemented with 2mM L-glutamine, 1mM pyruvate and 10 mM glucose, and cells were allowed to equilibrate at 37°C and 0% CO_2_ for 1 h. After calibration, oxygen consumption rate (OCR) was measured by the Seahorse XF96 Analyzer and recorded with XF Wave software 1.4.2. at 12 timepoints over the 80-min run: 3 times at basal respiration, 3 times after injection of 2.5 μM oligomycin to inhibit complex V, 3 times after injection of 2 μM of the ionophore carbonyl cyanide-p-trifluoromethoxyphenylhydrazone (FCCP) to depolarize the inner mitochondrial membrane, and 3 times after injection of 0.5 μM each of rotenone and antimycin A, to inhibit complexes I and III, respectively. After completion of the assay, cells were lysed in 1X RIPA buffer and total protein was quantified by bicinchoninic acid (BCA) assay. OCR values were normalized to the amount of protein per well and were used to calculate basal respiration, proton leak, maximal respiration, non-mitochondrial respiration, ATP production and spare respiratory capacity. Four independent Seahorse assay experiments were performed, with three wells per group per experiment.

**Cell culture and neurite growth**

Undifferentiated SH-SY5Y cells were used due to the reported effect of differentiating agents to decrease susceptibility of SH-SY5Y cells to PD-related insults (Cheung et al., 2009). Cells were grown in medium consisting of DMEM-F12 Ham (D6421) supplemented with 10% (v/v) foetal bovine serum (FBS), 1% (v/v) l-glutamine (G7513), 1% (v/v) penicillin/streptomycin (P4333) (all from Sigma). Cells were cultured at 37°C with 5% CO_2_. Cells were transfected using TransIT-X2® reagent according to the manufacturer’s instructions. For analysis of neurite growth, SH-SY5Y cells were co-transfected with 500 ng of pcDNA3-EGFP (Addgene plasmid # 13031; a gift from Doug Golenbock) along with 500ng of either pT-FLAG (Addgene plasmid #31385; a gift from Yegor Vassetzky (Dmitriev & Vassetzky, 2008) or FLAG-tagged SKOR1 (OriGene CAT#: RC234977) or in experiments involving alpha-synuclein overexpression, EGFP-alpha-synuclein-WT (Addgene plasmid # 40822 ; a gift from David Rubinsztein (FURLONG et al., 2000). Images of GFP-positive cells were captured for analysis of neurite growth at 72h post-transfection. Depending on the experiment, between 120-192 cells per experiment group from four independent experiments were imaged using an Olympus IX71 inverted microscope. For specific numbers in each experiment, see figure legend. Neurite growth was measured by opening each image in Image J, and manually tracing the neurite length of a given neurite using the trace function.

**Cell culture and BMP-SMAD signalling**

To assess BMP-SMAD signalling pathway activity in response to SKOR1 overexpression, a BMP-SMAD**-**GFP reporter assay was used. Briefly, cells were transfected using TransIT-X2® reagent according to the manufacturer’s instructions with either 250 ng of pT-FLAG (Addgene plasmid #31385; a gift from Yegor Vassetzky (Dmitriev & Vassetzky, 2008)) or FLAG-tagged SKOR1 (OriGene CAT#: RC234977), along with 500 ng of the Cignal GFP SMAD reporter plasmid (Qiagen, CCS-017G). Cells were imaged at 48h post-transfection, as recommended by the manufacturer, to quantify GFP fluorescence intensity as a readout of BMP-SMAD pathway-dependent transcription (Hegarty et al., 2014). Fluorescence intensities of 120 cells from 4 independent experiments were analysed using Image J software.

**LDH Assay**

A lactate dehydrogenase (LDH) assay was conducted following 72 h of treatment (3DIV) to determine cellular cytotoxicity levels by measuring LDH release in cellular media using CyQUANT LDH Cytotoxicity Assay (Thermo Fisher). Briefly, media was collected from 24-well plates and centrifuged at 1500 rpm for 5 min to remove any suspended cells. 50 µl of media was ten added per well to a clear, flat-bottomed 96-well plate, before adding an additional 50 µl of the assay substrate mix to each well and incubating in the dark at room temperature for 30 min. Following the incubation period, 50 µl of the assay stop solution was then added to each well before the absorbance in each well was measured at 490 nm and 680 nm using a Multiskan FC Microplate Photometer (Thermo Fisher).

**Immunocytochemical Staining of Cultured Cells**

For SH-SY5Y cells, the growth medium was first carefully removed from the 24-well plates. Wells were then carefully washed three times using 500 µl of 10 mM phosphate-buffered solution (PBS) for 2 min each, before adding 500 µl of 4% paraformaldehyde to each well and incubating at room temperature for 15 min to fix cells. Following fixation, cells were washed three times in 10mM PBS containing 0.02% Triton X-100 (PBS-T) for 5 min. Cells were then blocked by incubating for 1 h at room temperature in 500 µl of 5% bovine serum albumin (BSA) (Sigma) in PBS. Once blocked, the 5% BSA solution was removed and 200 µl of a solution containing the primary antibody diluted in 1% BSA in 10 mM PBS was added to the relevant wells, before incubating overnight at 4 °C. The primary antibodies used were rabbit anti-Lbxcor1 (Skor1) (1:500; Sigma #SAB2105374) and anti-phosphorylated-SMAD (pSMAD) 1/5/9 (1:500; Cell Signalling Technology #9516S). Following incubation, primary antibodies were removed, and cells were washed three times with PBS-T for 5 min each. Cells were then incubated in 200 µl of solution containing the appropriate Alexa Fluor 488, or 594 conjugated secondary antibodies (1:500; Invitrogen) diluted in 1% BSA in 10 mM PBS at room temperature, in the dark for 2 h. Following incubation, the secondary antibody solution was removed, and cells were washed three times with PBS-T for 5 min per wash. Cells were then incubated in 200 µl of solution containing 4'-6-Diamidino-2-phenylindole (DAPI) (1:3000; Sigma) in 10 mM PBS at room temperature for 5 min in the dark. Following DAPI incubation, cells were washed 3 more times in PBS for 5 min per wash, before a final addition of 500 µl of 10 mM PBS. Imageing was carried out on an inverted fluorescent microscope (IX71, Olympus), and quantification of fluorescence intensity in 192 cells from 4 independent experiments was carried out using Image J software.

**Statistical analysis**

Statistical analysis was performed using GraphPad Prism 9 (©2021 GraphPad Software, CA USA). All data are presented as the mean ± SEM of the number of experimental replicates rather than of individual cells. Statistical differences were analysed using Student’s *t*-test, one-way ANOVA, or two-way ANOVA as appropriate, with *post-hoc* tests as indicated in the figure legends.

**References**

Aranda, P.S., LaJoie, D.M., Jorcyk, C.L., 2012. Bleach gel: a simple agarose gel for analyzing RNA quality. Electrophoresis 33, 366-369.

Aranda, P.S., LaJoie, D.M., Jorcyk, C.L., 2012. Bleach gel: a simple agarose gel for analyzing RNA quality. Electrophoresis 33, 366-369.

Cheung, Y.T., Lau, W.K., Yu, M.S., Lai, C.S., Yeung, S.C., So, K.F., Chang, R.C., 2009. Effects of all-trans-retinoic acid on human SH-SY5Y neuroblastoma as in vitro model in neurotoxicity research. Neurotoxicology 30, 127-135.

Dowd, E., Monville, C., Torres, E.M., Dunnett, S.B., 2005. The Corridor Task: a simple test of lateralised response selection sensitive to unilateral dopamine deafferentation and graft-derived dopamine replacement in the striatum. Brain Res Bull 68, 24-30.

FURLONG, R.A., NARAIN, Y., RANKIN, J., WYTTENBACH, A., RUBINSZTEIN, D.C., 2000. α-Synuclein overexpression promotes aggregation of mutant huntingtin. Biochemical Journal 346, 577-581.

Hegarty, S.V., Collins, L.M., Gavin, A.M., Roche, S.L., Wyatt, S.L., Sullivan, A.M., O'Keeffe, G.W., 2014. Canonical BMP-Smad signalling promotes neurite growth in rat midbrain dopaminergic neurons. Neuromolecular Med 16, 473-489.

McCarthy, E., Barron, A., Morales-Prieto, N., Mazzocchi, M., McCarthy, C.M., Collins, L.M., Sullivan, A.M., O'Keeffe, G.W., 2022. Gene Co-expression Analysis of the Human Substantia Nigra Identifies ZNHIT1 as an SNCA Co-expressed Gene that Protects Against alpha-Synuclein-Induced Impairments in Neurite Growth and Mitochondrial Dysfunction in SH-SY5Y Cells. Mol Neurobiol.

Naughton, C., O'Toole, D., Kirik, D., Dowd, E., 2017. Interaction between subclinical doses of the Parkinson's disease associated gene, alpha-synuclein, and the pesticide, rotenone, precipitates motor dysfunction and nigrostriatal neurodegeneration in rats. Behav Brain Res 316, 160-168.

Negrini, M., Tomasello, G., Davidsson, M., Fenyi, A., Adant, C., Hauser, S., Espa, E., Gubinelli, F., Manfredsson, F.P., Melki, R., Heuer, A., 2022. Sequential or Simultaneous Injection of Preformed Fibrils and AAV Overexpression of Alpha-Synuclein Are Equipotent in Producing Relevant Pathology and Behavioral Deficits. J Parkinsons Dis 12, 1133-1153.

Olsson, M., Nikkhah, G., Bentlage, C., Bjorklund, A., 1995. Forelimb akinesia in the rat Parkinson model: differential effects of dopamine agonists and nigral transplants as assessed by a new stepping test. J Neurosci 15, 3863-3875.
