# Supplementary material for: Human α‐synuclein overexpression upregulates SKOR1 in a rat model of simulated nigrostriatal ageing
Source: Aging Cell. 2024 Mar 26;23(6):e14155. doi: 10.1111/acel.14155 (PMC11296121; doi:10.1111/acel.14155)
Supplement: Supplementary file 3 — Data S2. [file ACEL-23-e14155-s003.docx]

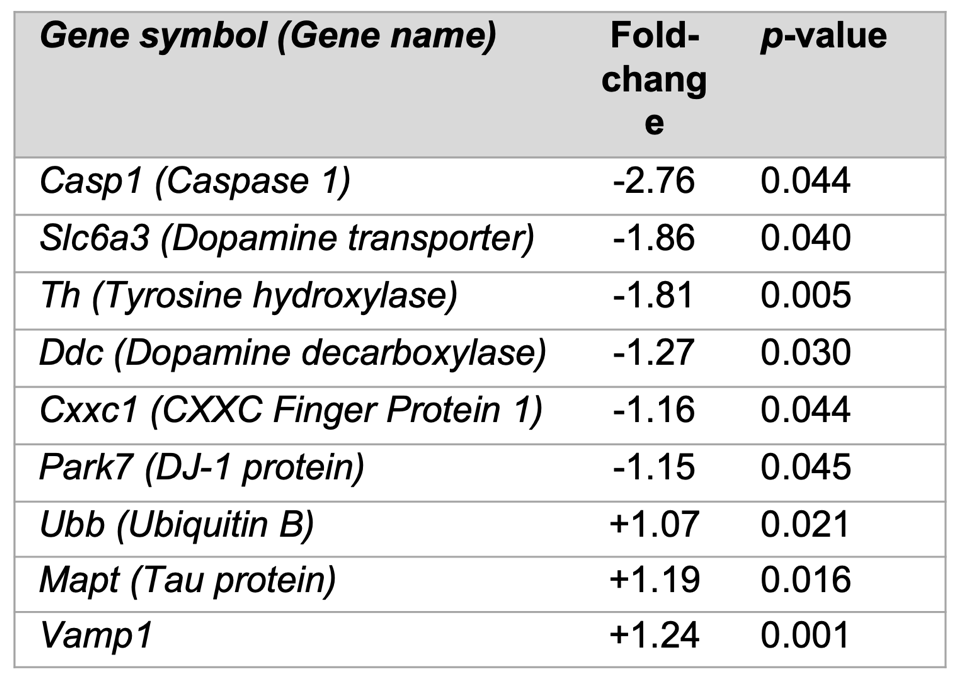


**Supplementary Figure S1: List of genes from the Parkinson’s disease RT2 Profiler PCR Array that were differentially expressed in the AAV-aSyn group.** A (-) symbol denotes reduced expression and (+) symbol denotes overexpression in the AAV-aSyn group.
